# A Wirelessly Powered 4-Channel Neurostimulator for Reconstructing Walking Trajectory

**DOI:** 10.3390/s22197198

**Published:** 2022-09-22

**Authors:** Masaru Takeuchi, Katsuhiro Tokutake, Keita Watanabe, Naoyuki Ito, Tadayoshi Aoyama, Sota Saeki, Shigeru Kurimoto, Hitoshi Hirata, Yasuhisa Hasegawa

**Affiliations:** 1 Department of Micro-Nano Mechanical Science and Engineering, Nagoya University, Nagoya 464-8601, Japan; 2 Department of Human Enhancement and Hand Surgery, Nagoya University, Nagoya 464-8601, Japan

**Keywords:** neurostimulation, wireless powering, implantable device

## Abstract

A wirelessly powered four-channel neurostimulator was developed for applying selective Functional Electrical Stimulation (FES) to four peripheral nerves to control the ankle and knee joints of a rat. The power of the neurostimulator was wirelessly supplied from a transmitter device, and the four nerves were connected to the receiver device, which controlled the ankle and knee joints in the rat. The receiver device had functions to detect the frequency of the transmitter signal from the transmitter coil. The stimulation site of the nerves was selected according to the frequency of the transmitter signal. The rat toe position was controlled by changing the angles of the ankle and knee joints. The joint angles were controlled by the stimulation current applied to each nerve independently. The stimulation currents were adjusted by the Proportional Integral Differential (PID) and feed-forward control method through a visual feedback control system, and the walking trajectory of a rat’s hind leg was reconstructed. This study contributes to controlling the multiple joints of a leg and reconstructing functional motions such as walking using the robotic control technology.

## 1. Introduction

In the most recent decade, implantable devices for functional electrical stimulation (FES) have been actively studied for reconstructing functional motions of patients who had a spinal cord injury and consequently lost their functional movements of limbs. For example, Ajiboye et al. developed the FES system using a Brain–Computer Interface (BCI) to regenerate limb motions using FES applied to peripheral muscles and nerves [1]. Bouton also conducted the FES through the neuromuscular electrical stimulation sleeve to reconstruct functional motions of limbs in a paralyzed patient [2]. Memberg et al. implanted neuroprostheses to restore arm and hand function of humans for more than 7 years [3]. In these studies, the muscle motion could be generated by the FES after the spinal injury because the lower motor neurons had kept their characteristics and still maintained excitability to the electrical stimulation [4]. Thus, the FES is one of the effective treatments for patients with spinal cord injury to reconstruct the functional motion of their limbs.

On the other hand, peripheral nerve disconnections cannot be used in FES treatment. The peripheral nerve injury causes paralysis of limbs because of severe muscle atrophy. For example, amyotrophic lateral sclerosis (ALS) is one of the serious diseases to show muscle atrophy because of peripheral motor nerve dysfunctions. In such a disease, the lower motor neurons cannot maintain the ability to respond to electrical stimulation. Therefore, FES cannot be an effective treatment for those diseases. To solve the limitation and prevent the degradation of denervated muscles and restore muscle activities, FES treatment after the transplantation of motor neurons onto the damaged peripheral nerves has been developed [5,6]. Recovery of muscle activity has been found by FES applied to the peripheral nerves after injection of motor neurons into the sciatic nerve of a rat. Muscle atrophy was prevented by applying a motor neuron injection and FES even if the muscle was denervated [7,8].

For clinical use of such FES technologies to stimulate the peripheral nerve and restore functional motion of the limbs, wirelessly powered implantable devices are desired because the implantable devices prevent the risk of infection by wiring. Implantable FES devices used to electrically stimulate peripheral nerves have been recently and actively developed, and some devices have been used in humans clinically, such as Vagus Nerve Stimulation (VNS) to treat epilepsy, and artificial cochlea [9,10,11,12], as the implanted device can precisely control the stimulation site, although it is invasive compared with surface stimulation of muscles using surface electrodes on skin. A feedback control system was employed in some implantable FES devices, such as Deep Brain Stimulation (DBS) systems [13,14,15], VNS systems [16,17], and neuroprosthetic systems [18]. Hence, robotics technologies have an important role in controlling and reconstructing functional motions of legs or hands [19]. We proposed a multiple-channel neurostimulator to stimulate peripheral nerves with visual feedback control to achieve control of limb motion [20] as a new treatment strategy for patients of ALS and peripheral nerve injuries. In our previous study, a two-channel wirelessly powered neurostimulator was developed, and a rat ankle joint was controlled by FES to multiple peripheral nerves through the visual feedback control method [20,21]. In this study, we developed a four-channel wirelessly powered neurostimulator to control two joints in a rat: ankle and knee joints. The device we developed in this study has about 40 times higher resolution of stimulation current (from 0 to 0.4 mA with 0.1 μA resolution) compared with our previous study [21], the stimulation frequency is controllable from 0 to 1 kHz, and duration is also controllable from 0 to 1 ms. The proposed method is a new strategy to reconstruct functional motion using their own hands or legs like robotic prostheses [22,23].

## 2. Materials and Methods

### 2.1. Animals

All animal experiments and procedures used in this study were approved by the Animal Ethics Research Committee at Nagoya University (registration number M210152-004). Three adult Fischer 344 rats (18 to 20-week-old, weight 320 to 350 g) (Japan SLC, Shizuoka, Japan) were used: one was for validating selective stimulation of four nerves for generating dorsal/plantar flexion of the ankle joint and flexion/extension of the knee joint (see results in Section 3.1), another was for visual feedback control, and the other was for feedback + feedforward control (see results in Section 3.2). The rats were under anesthesia (O${}_{2}$ was 0.6 L/min, Isoflurane was 3 to 4% at the start of the anesthesia, and 2% to maintain the anesthesia) in all experiments to prevent voluntary motion.

### 2.2. Four-Channel Neurostimulator

**Figure 1:**
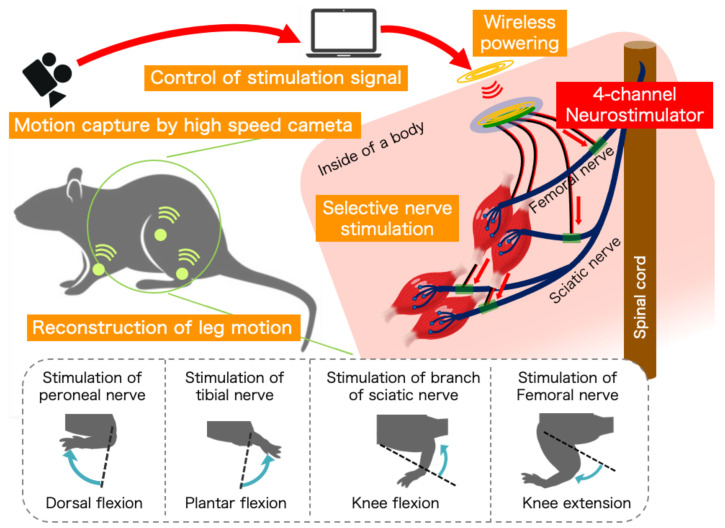
A schematic of the concept of the multi-channel neurostimulator to stimulate peripheral nerves using visual feedback control to reconstruct functional motions; rat leg motion can be controlled by changing the stimulated nerve, and the stimulation can be controlled by a visual feedback system using a high-speed camera.

In this study, a wirelessly powered four-channel neurostimulator was developed to conduct FES for four nerves in a rat selectively to generate ankle and knee motions, as shown in Figure 1: the tibial and peroneal nerves were related to ankle joint motion and the femoral nerve and branch of sciatic nerve connected to femoral biceps were related to knee joint motion. A magnetic resonance method [24,25] was employed to conduct wireless powering to the neurostimulator, which was developed based on our previous study [20,21].

#### 2.2.1. Transmitter Circuit for Four-Channel Neurostimulator

**Figure 2:**
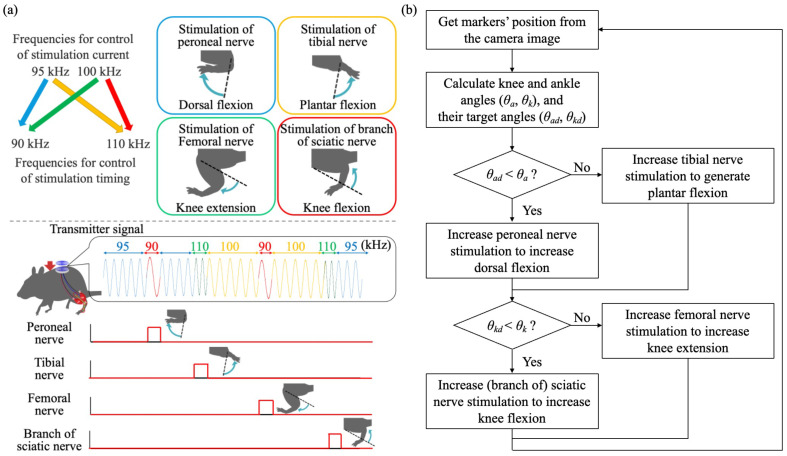
A schematic indicating the selective stimulation of the rat’s four nerves to generate ankle and knee joint motions by switching the frequency of the transmitter signal. (**a**) A schematic of switching the frequency of the transmitter signal to generate four different motions to the rat’s hind leg. (**b**) Flowchart of nerve stimulation for 2 joint control.

In our newly developed four-channel device, four different frequencies, namely, 90 kHz, 95 kHz, 100 kHz, and 110 kHz, were used to send information about which nerve should be stimulated with sending power from a transmitter device to a receiver device by a magnetic resonance method, as shown in Figure 2a. The receiver device detected the frequency and selected the stimulation nerve from the four nerves. If the frequency changed from 100 kHz to 90 kHz, the peroneal nerve was stimulated to generate dorsal flexion, while the tibial nerve was stimulated to generate plantar flexion when the frequency was changed from 100 kHz to 110 kHz. A frequency of 100 kHz was used to stimulate neither the peroneal nerve nor the tibial nerve. Thus, the ankle joint was controlled by changing the transmitter frequency from 100 kHz to 90 kHz or 110 kHz.

To control the knee joints, we also used other pairs of transmitter frequencies. If the frequency was changed from 95 kHz to 90 kHz, the branch of the sciatic nerve connected to the femoral biceps was stimulated to generate knee flexion, while the femoral nerve was stimulated when the frequency was changed from 95 kHz to 110 kHz. A frequency of 95 kHz was used to stimulate neither the sciatic nerve nor the femoral nerve. Thus, the knee joint was controlled by changing the transmitter frequency from 95 kHz to 90 kHz or 110 kHz. The stimulation of four nerves was conducted by comparing present knee/ankle angles and target knee/ankle angles, as shown in Figure 2b.

**Figure 3:**
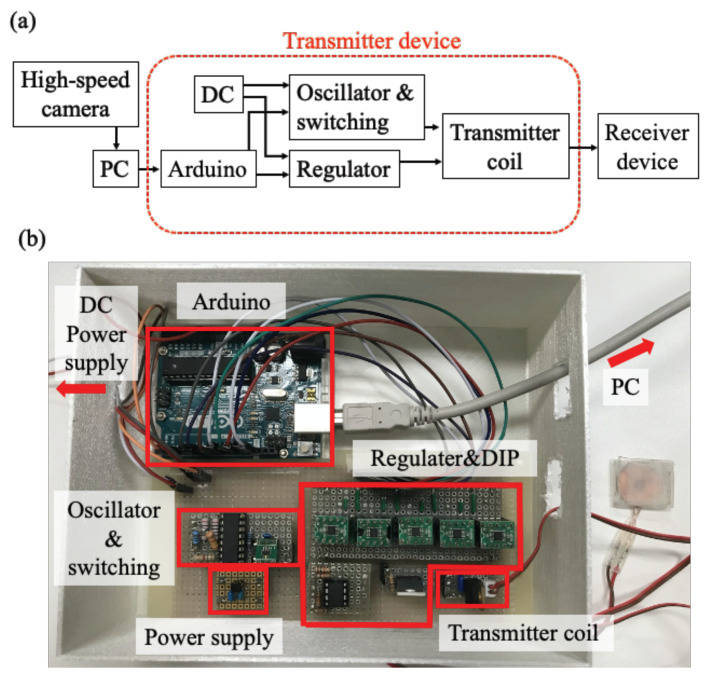
A transmitter device system for a wirelessly powered four-channel neurostimulator. (**a**) A schematic of the transmitter device system; (**b**) the fabricated transmitter device.

In this study, rat ankle and knee angles were controlled using the developed four-channel neurostimulator with visual feedback control. The transmitter device, which was used to send a control signal to the receiver device for visual feedback control of the rat ankle and knee angles, was prepared, as shown in Figure 3. Figure 3a indicates the information flow of the transmitter device. The transmitter device had a microcomputer to receive information from a PC about which nerve should be stimulated and how much current should be applied to the target nerve. An oscillator and switching circuit received the information from the microcomputer and controlled the frequency of the transmitter signal, as the frequency was used to send information to the receiver device about the selection of stimulating a nerve. A voltage regulator adjusted the amplitude of the transmitter signal by the output from the microcomputer to send information about the stimulation current to the receiver device. Figure 3b is the fabricated transmitter device. The transmitter coil in the device was 18 mm in diameter, 26 μF self-inductance, and 25 quality factors.

#### 2.2.2. Receiver Circuit for Four-Channel Neurostimulator

**Figure 4:**
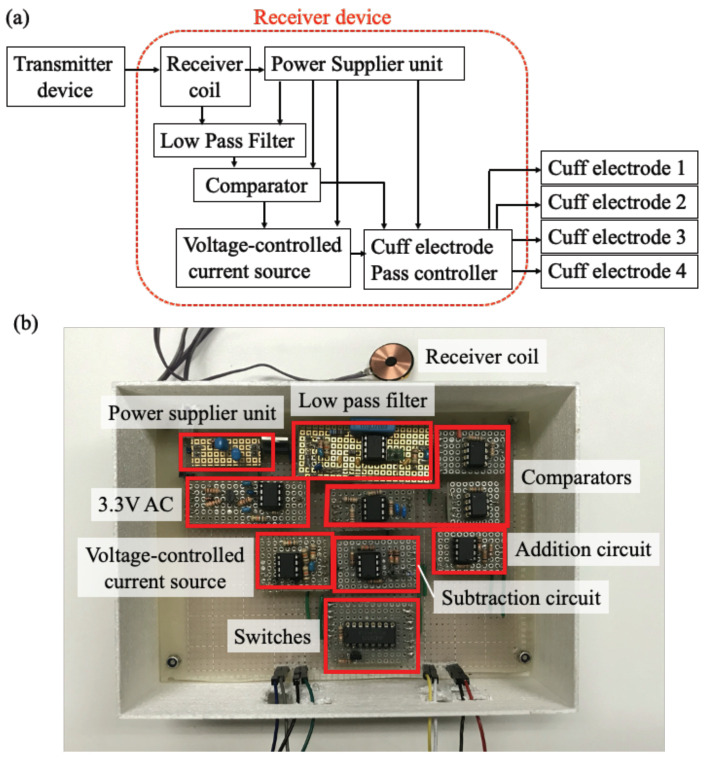
A receiver device for a wirelessly powered four-channel neurostimulator. (**a**) A schematic of the receiver device system; (**b**) the fabricated receiver device.

Figure 4 explains the information flow of the receiver circuit and fabricated receiver device. Figure 4a indicates the system flow of the receiver device. The device had a receiver coil to receive power from a transmitter coil. A low pass filter and comparators were used to detect which frequency was sent from the transmitter coil. In the device, three comparators were employed: the first comparator was “on” when the frequency of the transmitter signal was more than 110 kHz, the second one was “on” when the frequency was less than 90 kHz, and the third one was “on” when the frequency was more than 100 kHz. The third comparator combined with a delay circuit and could stay in “on” condition for 1 ms even if the frequency of the transmitter signal became less than 100 kHz. Therefore, the third comparator and the first or second comparator could be kept in “on” condition simultaneously. To switch the output electrode, a combination of two signals was used: one was the output from an adder circuit of the first and second comparators, and the other was the output from the third comparator.

A voltage-controlled current source was installed to achieve control of stimulation current to the nerves. The receiver device had four stimulation electrodes, and the stimulation electrode and stimulation current were selected from the transmitter signal by the switches. The receiver coil was the same as the transmitter coil, which had 18 mm in diameter, with 26 μF self-inductance and a quality factor 25. Figure 4b shows the fabricated receiver device. The device generated 0 to 0.4 mA stimulation current with 0.1 μA resolution, which was about 40 times higher resolution compared with our previous study [21]. The switching from one stimulation channel to another required less than 2 ms, and the frequency of stimulation current was controllable up to 1 kHz. The duration of the stimulation current was also controllable from 0 to 1 ms. In this study, the receiver device was fabricated to put outside the body to check whether the electrical circuit worked as we designed. However, the receiver device was designed to receive power wirelessly, and it can be minimized into an implantable size in the near future.

### 2.3. Selective Stimulation of Four Nerves in a Rat

The cuff electrodes we developed previously [8,21] were placed onto the rat’s peroneal nerve and tibial nerve to generate dorsal and plantar flexion of the ankle joint, respectively, and also connected to the branch of the sciatic nerve (connected to femoral biceps) and the femoral nerve to generate knee flexion and extension, respectively. The stimulation signal of nerves was set at 50 Hz to generate tetanic contraction of muscles, and its duration was set at 0.2 ms. The stimulation current to the tibial nerve, peroneal nerve, branch of the sciatic nerve, and the femoral nerve was all set at 0.40 mA to observe the four motions in two joints: dorsal/planter of the ankle joint and flexion/extension of the knee joint. The receiver device was wirelessly powered by the transmitter device. The stimulation of the tibial nerve, peroneal nerve, branch of the sciatic nerve, and femoral nerve were conducted in order.

### 2.4. Visual Feedback Control of Two Joints in a Rat

**Figure 5:**
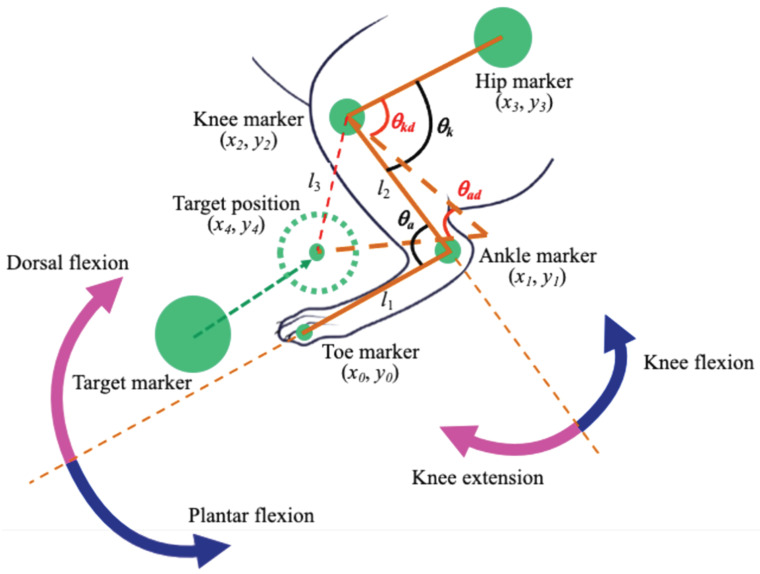
Experimental setup for visual feedback control of the rat knee and ankle joint angles.

To conduct control of the rat knee and ankle joint angles using a high-speed camera, the current position of the rat leg has to be detected through the camera. We used the same experimental setup in our previous study [8,21]. A camera (MQ003CG-CM, Ximea, Münster, Germany) was used to detect the angles of the knee and ankle joints using markers on a rat. The markers were fabricated using a 3D printer, and the green color was painted to be identified by the image processing. Four markers were placed on the rat’s toe, ankle, knee, and hip, respectively. Another maker was moved by hand to be used as a target marker to indicate the target position of a rat’s toe. Figure 5 shows the experimental setup to detect the ankle and knee joint angles from the markers on a rat.

In our experimental setup, the different sizes of markers were prepared to distinguish the position of the hip, knee, ankle, and tow of the rat. The largest marker was used as the target marker, and markers on the hip, knee, ankle, and toe became smaller in this order. The target marker was used to indicate the target position of the rat toe. To prevent overlapping of the target marker and the toe marker in the camera image, the target position of the rat toe was sifted by arbitrary pixels in the *x* and *y* axis in the camera monitor from the target marker, as shown in Figure 5. From these markers on the rat leg, the hip position ($x_{3}$, $y_{3}$), knee position ($x_{2}$, $y_{2}$), ankle position ($x_{1}$, $y_{1}$), and toe position ($x_{0}$, $y_{0}$) were detected. The angle of the ankle joint $\theta_{a}$ and knee joint $\theta_{k}$ were calculated using Equations (1) and (2), respectively:

(1) $$\theta_{a}=tan^{-1}(\frac{y_{0}-y_{1}}{x_{0}-x_{1}})-tan^{-1}(\frac{y_{2}-y_{1}}{x_{2}-x_{1}})\;$$

(2) $$\theta_{k}=tan^{-1}(\frac{y_{3}-y_{2}}{x_{3}-x_{2}})-tan^{-1}(\frac{y_{1}-y_{2}}{x_{1}-x_{2}})\;$$

The target angle of ankle joint $\theta_{ad}$ was calculated by Equation (3):

(3) $$\theta_{ad}=cos^{-1}(\frac{l_{1}^{2}+l_{2}^{2}-l_{3}^{2}}{2l_{1}l_{2}})\;$$

where $l_{1}$, $l_{2}$, and $l_{3}$ were defined as Equations (4)–(6):

(4) $$l_{1}=\sqrt{{\left(x_{0}-x_{1}\right)}^{2}+{\left(y_{0}-y_{1}\right)}^{2}}\;$$

(5) $$l_{2}=\sqrt{{\left(x_{1}-x_{2}\right)}^{2}+{\left(y_{1}-y_{2}\right)}^{2}}\;$$

(6) $$l_{3}=\sqrt{{\left(x_{2}-x_{4}\right)}^{2}+{\left(y_{2}-y_{4}\right)}^{2}}\;$$

The target angle of knee joint $\theta_{kd}$ was calculated from the position of the target marker ($x_{4}$, $y_{4}$) in Equation (7):

$$\theta_{kd}=tan^{-1}(\frac{y_{3}-y_{2}}{x_{3}-x_{2}})-tan^{-1}(\frac{y_{0}-y_{2}}{x_{0}-x_{2}})$$

(7) $$-cos^{-1}(\frac{l_{2}^{2}+l_{3}^{2}-l_{1}^{2}}{2l_{2}l_{3}})\;$$

The stimulation currents to the four nerves were controlled using the Proportional Integral Differential (PID) control method to change ankle angle $\theta_{a}$ and knee angle $\theta_{k}$ for moving the rat toe position ($x_{0}$, $y_{0}$) to be the same as the target position ($x_{4}$, $y_{4}$). The plantar flexion of the ankle can be generated by the stimulation of the tibial nerve, and the dorsal flexion of the ankle can be generated by the stimulation of the peroneal nerve. The knee flexion can be generated by stimulating the branch of the sciatic nerve connected to the femoral biceps, and the knee extension can be generated by stimulating the femoral nerve.

When the ankle angle is larger than the target angle of the ankle ($\theta_{ad}$<$\theta_{a}$), dorsal flexion should be generated. The stimulation current to the peroneal nerve at the time step *k* ($S^{p}\left(k\right)$) was calculated using Equations (8)–(11), as shown below [21]:

(8) $$S_{p}^{p}\left(k\right)=K_{p}\left(\theta_{a}\left(k\right)-\theta_{ad}\left(k\right)\right)\;$$

(9) $$S_{i}^{p}\left(k\right)=S_{i}^{p}\left(k-1\right)+K_{i}\left(\theta_{a}\left(k\right)-\theta_{ad}\left(k\right)\right)\;$$

(10) $$S_{d}^{p}\left(k\right)=K_{d}\left(\theta_{a}\left(k\right)-\theta_{ad}\left(k\right)\right)-\left(\theta_{a}\left(k-1\right)-\theta_{ad}\left(k-1\right)\right)\;$$

(11) $$S^{p}\left(k\right)=S_{p}^{p}\left(k\right)+S_{i}^{p}\left(k\right)+S_{d}^{p}\left(k\right)\;$$

where $S_{p}^{p}\left(k\right)$ is the stimulation current at time step *k* from the Proportional (P) control, $S_{i}^{p}\left(k\right)$ is the current from the Integral (I) control, and $S_{d}^{p}\left(k\right)$ is the current from the Differential (D) control. $K_{p}$, $K_{i}$, and $K_{d}$ are constant coefficients of the P, I, and D control, respectively.

When $\theta_{ad}$≥$\theta_{a}$, the plantar flexion of the rat ankle joint should be generated. The stimulation current to the tibial nerve at the time step *k* ($S^{t}\left(k\right)$) was calculated by Equations (12)–(15):

(12) $$S_{p}^{t}\left(k\right)=K_{p}\left(\theta_{a}\left(k\right)-\theta_{ad}\left(k\right)\right)\;$$

(13) $$S_{i}^{t}\left(k\right)=S_{i}^{t}\left(k-1\right)+K_{i}\left(\theta_{a}\left(k\right)-\theta_{ad}\left(k\right)\right)\;$$

(14) $$S_{d}^{t}\left(k\right)=K_{d}\left(\theta_{a}\left(k\right)-\theta_{ad}\left(k\right)\right)-\left(\theta_{a}\left(k-1\right)-\theta_{ad}\left(k-1\right)\right)\;$$

(15) $$S^{t}\left(k\right)=S_{p}^{t}\left(k\right)+S_{i}^{t}\left(k\right)+S_{d}^{t}\left(k\right)\;$$

where $S_{p}^{t}\left(k\right)$ is the stimulation current to the tibial nerve at time step k controlled by the P control, $S_{i}^{t}\left(k\right)$ is the current controlled by the I control, and $S_{d}^{t}\left(k\right)$ is the current controlled by the D control. The total stimulation current to the tibial nerve $S_{t}\left(k\right)$ was defined as the summation of $S_{p}^{t}\left(k\right)$, $S_{i}^{t}\left(k\right)$, and $S_{d}^{t}\left(k\right)$.

In the same procedure, the knee flexion and extension were controlled by adjusting the stimulation current to the branch of the sciatic nerve connected to the femoral biceps and the femoral nerve. When the knee joint angle is larger than the target angle of the knee ($\theta_{kd}$<$\theta_{k}$), knee flexion should be generated. The stimulation current to the branch of the sciatic nerve connected to the femoral biceps at the time step *k* ($S_{s}\left(k\right)$) was calculated using Equations (16)–(19):

(16) $$S_{p}^{s}\left(k\right)=K_{p}\left(\theta_{k}\left(k\right)-\theta_{kd}\left(k\right)\right)\;$$

(17) $$S_{i}^{s}\left(k\right)=S_{i}^{s}\left(k-1\right)+K_{i}\left(\theta_{k}\left(k\right)-\theta_{kd}\left(k\right)\right)\;$$

(18) $$S_{d}^{s}\left(k\right)=K_{d}\left(\theta_{k}\left(k\right)-\theta_{kd}\left(k\right)\right)-\left(\theta_{k}\left(k-1\right)-\theta_{kd}\left(k-1\right)\right)\;$$

(19) $$S^{s}\left(k\right)=S_{p}^{s}\left(k\right)+S_{i}^{s}\left(k\right)+S_{d}^{s}\left(k\right)\;$$

where $S_{p}^{s}\left(k\right)$ is the stimulation current to the branch of the sciatic nerve (connected to the femoral biceps) at time step k from the P control, $S_{i}^{s}\left(k\right)$ is the current from the I control, and $S_{d}^{s}\left(k\right)$ is the current from the D control. In the case of $\theta_{kd}$≥$\theta_{k}$, the femoral nerve should be stimulated to generate knee extension. The stimulation current to the femoral nerve at the time step *k* ($S_{f}\left(k\right)$) was calculated using Equations (20)–(23) as shown below:

(20) $$S_{p}^{f}\left(k\right)=K_{p}\left(\theta_{kd}\left(k\right)-\theta_{k}\left(k\right)\right)\;$$

(21) $$S_{i}^{f}\left(k\right)=S_{i}^{f}\left(k-1\right)+K_{i}\left(\theta_{kd}\left(k\right)-\theta_{kd}\left(k\right)\right)\;$$

(22) $$S_{d}^{f}\left(k\right)=K_{d}\left(\theta_{k}\left(k\right)-\theta_{kd}\left(k\right)\right)-\left(\theta_{k}\left(k-1\right)-\theta_{kd}\left(k-1\right)\right)\;$$

(23) $$S^{f}\left(k\right)=S_{p}^{f}\left(k\right)+S_{i}^{f}\left(k\right)+S_{d}^{f}\left(k\right)\;$$

where $S_{p}^{f}\left(k\right)$ is the stimulation current to the femoral nerve at time step *k* from the P control, $S_{i}^{f}\left(k\right)$ is the current from the I control, and $S_{d}^{f}\left(k\right)$ is the current from the I control.

**Figure 6:**
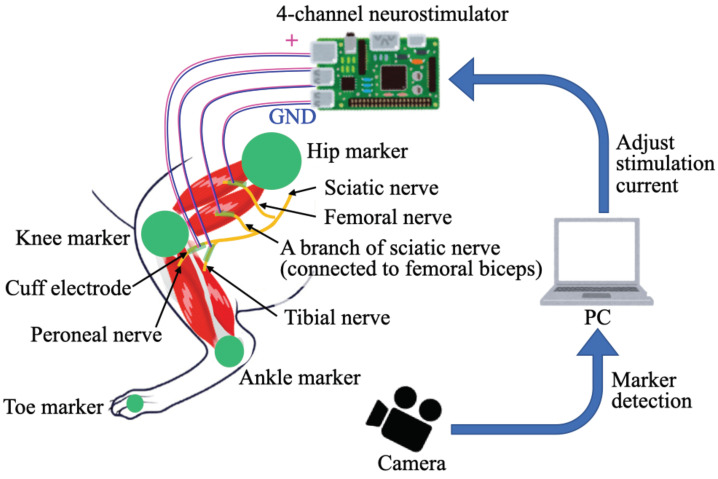
A schematic of the experimental system for visual feedback control of ankle and knee joints using a four-channel neurostimulator.

Figure 6 shows the visual feedback control of the ankle and knee angles in the experimental system. The rat was under anesthesia during the experiment to prevent the voluntary motion of the rat. The cuff electrodes we developed in the previous study [21] were used as interfaces between the peripheral nerves and the four-channel neurostimulator. Cuff electrodes were one of the important interfaces to connect nerves and machines and have been actively developed and applied to stimulate nerves in humans or animals [26]. The two cuff electrodes from the four-channel neurostimulator were connected to the tibial and peroneal nerves, respectively, to control the dorsal/plantar flexion of the ankle joint. The other two cuff electrodes from the four-channel neurostimulator were connected to the branch of the sciatic nerve (connected to femoral biceps) and femoral nerve, respectively, for controlling flexion/extension of the knee joint. Hence, the stimulation current was applied to the nerves through the four-channel neurostimulator. The high-speed camera obtained images at 250 Hz, and the stimulation current was updated at 12 Hz from the PID controller of the visual feedback system. This 12 Hz updating frequency of the stimulation current was caused by the limitation of the speed of the serial communication from the PC to the microcomputer (Arduino Uno R3; open-source hardware, and Arduino IDE 1.8.19, open-source software, Interaction Design Institute Ivrea, Ivrea, Italy) in our system. The transmitter system applied 2.25 W (0.25 A, 9.0 V) with a coil gap of 2.2 mm to use the receiver device by wireless powering in the experiments.

## 3. Results

### 3.1. Selective Stimulation of Four Nerves in a Rat

**Figure 7:**
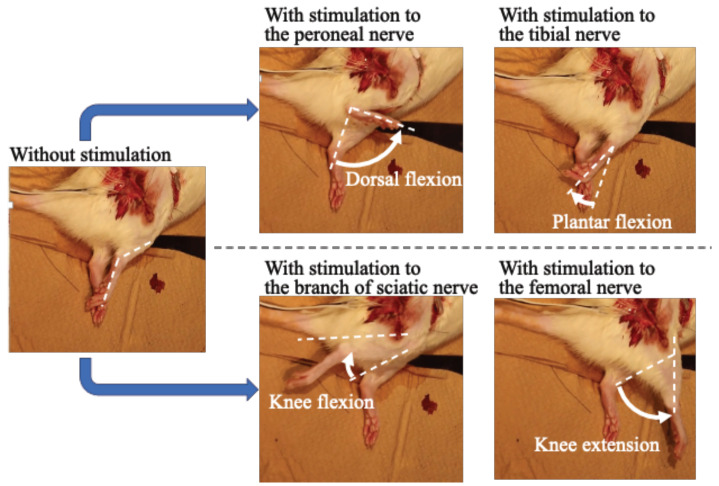
Selective stimulation of rat nerves for generating dorsal/plantar flexion of the ankle joint and flexion/extension of the knee joint.

The experimental results of stimulating four nerves in order are shown in Figure 7. Thus, the plantar/dorsal flexion of the ankle joint and flexion/extension of the knee joint were observed when the stimulation to the tibial nerve, peroneal nerve, branch of the sciatic nerve, and femoral nerve were conducted, respectively. Hence, the results showed that the developed four-channel neurostimulator could generate dorsal/plantar flexion of a rat ankle joint and flexion/extension of a rat knee joint by design.

### 3.2. Visual Feedback Control of Rat Ankle and Knee Joints

In the visual feedback experiments, the five markers (target, hip, knee, ankle, and toe) were captured by the high-speed camera. Their positions were detected using the color space of the Hue, Saturation, Value (HSV) model. In our system, each HSV parameter had a number from 0 to 255. The four threshold values, minimum hue, maximum hue, saturation, and the value, were adjusted in the experiments because those parameters were changed by the lighting condition of the experimental space. In the experiment, the minimum hue was set at 0, the maximum hue was 71, the saturation was 12, and the value was set at 0.

The range of stimulation current to the four nerves was checked before starting the visual feedback experiment. At first, the stimulation current of the peroneal nerve was gradually increased, and the minimum stimulation current of the peroneal nerve $S_{min}^{p}$ was found when the ankle leg started towards dorsal flexion. Then, the stimulation current was gradually increased, and the maximum stimulation current of the perineal nerve $S_{max}^{p}$ was found when the ankle became the maximum angle of dorsal flexion. In the same procedures, the range of the stimulation current was found for the tibial nerve $S_{min}^{t}$ and $S_{max}^{t}$, for the branch of the sciatic nerve $S_{min}^{s}$ and $S_{max}^{s}$, and for the femoral nerve $S_{min}^{f}$ and $S_{max}^{f}$, respectively. In our experimental setup, each value was fixed as follows: $S_{min}^{p}$ = 81 μA, $S_{max}^{p}$ = 354 μA, $S_{min}^{t}$ = 117 μA, $S_{max}^{t}$ = 156 μA, $S_{min}^{s}$ = 81 μA, $S_{max}^{s}$ = 90 μA, $S_{min}^{f}$ = 58 μA, and $S_{max}^{f}$ = 100 μA. The stimulation currents in each nerve were controlled in the range.

After the range of stimulation currents was fixed, the visual feedback control of the rat ankle and knee joints was tested using the four-channel neurostimulator. The target position of the rat toe was changed from the target marker by adding 620 pixels on the *x*-axis and 481 pixels on the *y*-axis in the image of a high-speed camera. The target marker was moved manually, and the stimulation currents to the four nerves were adjusted independently using the PID controller. The parameters for PID control were set experimentally in the procedures as follows: at first, $K_{p}$, $K_{i}$, and $K_{d}$ were set at 0, and $K_{p}$ was gradually increased until oscillation of the ankle or knee joint was observed, and the maximum value without leg oscillation was fixed as the $K_{p}$. Then, $K_{i}$ was gradually increased until oscillation of the ankle or knee joint was observed, and the maximum value without leg oscillation was fixed as the $K_{i}$. Finally, $K_{d}$ was gradually increased and fixed just before the generation of the leg oscillation. In the experiment, the parameters were set to the values of $K_{p}$ = 1, $K_{i}$ = 0.7, and $K_{d}$ = 0. The transmitter system used 0.25 A, 9.0 V, and 2.25 W with a coil gap of 2.2 mm for wireless powering. The rat was under anesthesia (O${}_{2}$ was 0.6 L/min, Isoflurane was 3 to 4% at the start of the anesthesia, and 2% to maintain the anesthesia) during the experiment.

**Figure 8:**
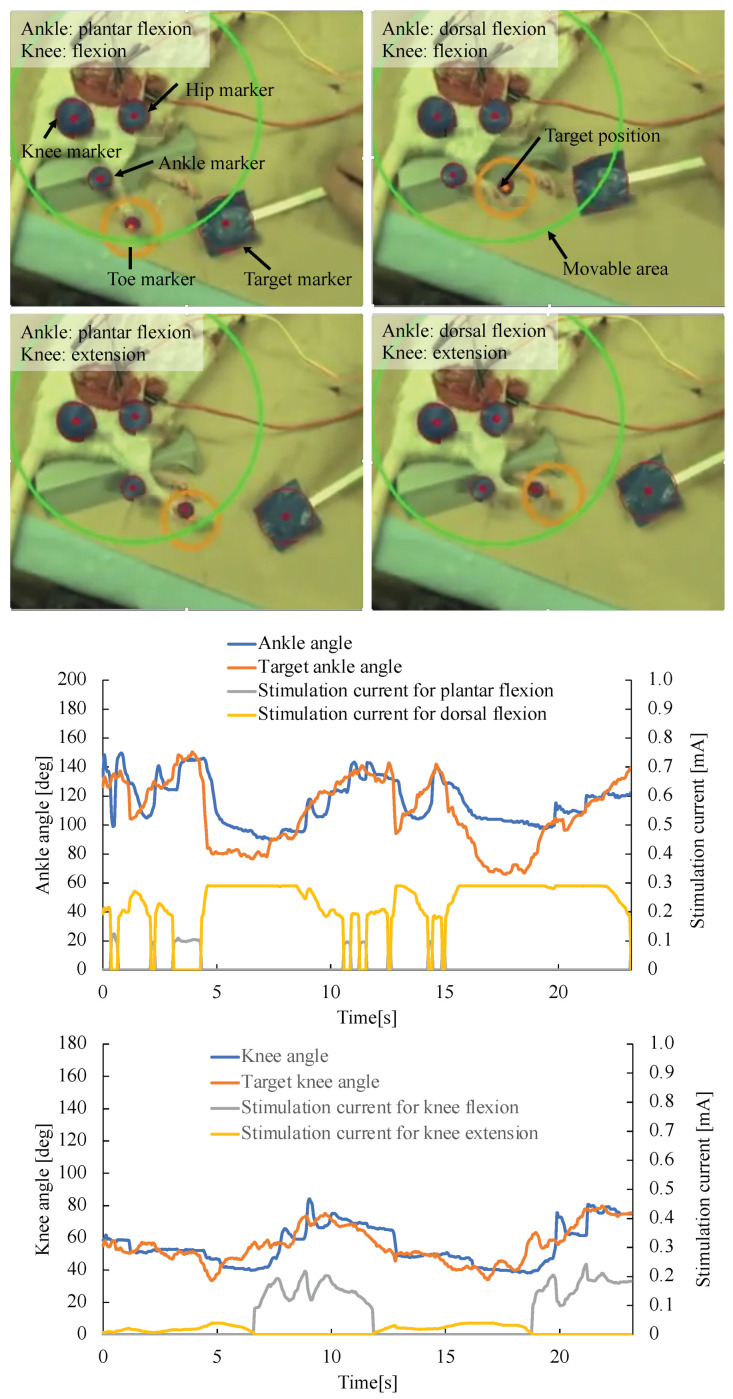
Experimental results about visual feedback control of rat knee and ankle joints. The lower two graphs indicate joint angle, target joint angle, and stimulation currents to the nerves during the experiment.

Figure 8 shows the experimental results when the ankle and knee joints were largely moved. In the experiment, the target marker was moved manually by observing the monitor, which indicated the target position of the toe with an orange circle and the limitation of a movable area with the green circle. The movable area was determined from the lengths $l_{1}$ and $l_{2}$ in Figure 5. If the target position of the rat toe was moved to the outside of the movable area, the nerve stimulation signal was not renewed, and the previous stimulation signal was applied to the nerves. When the target position was in front of the leg and close to the rat body, the ankle joint showed dorsal flexion, and the knee joint showed extension, as shown in the lower-right photo in Figure 8. When the marker was moved behind the leg, the knee joint was changed to flexion, as shown in the upper-right photo in Figure 8. The ankle joint became plantar flexion when the target position was moved far from the rat body, as shown in two photos of the left side in Figure 8. The graphs indicate the motion of ankle and knee joints in the experiment. Thus, the rat toe position was controlled in the range of 92 to 152 degrees at the ankle joint and 40 to 79 degrees at the knee joint by changing the angles of the rat ankle and knee joints. However, the motion control should be faster to reconstruct functional motions such as walking.

To improve the control response of the rat leg and reconstruct a walking motion, feed-forward control was added. The stimulation current to the peroneal nerve at time step *k* by feed-forward control $S_{ff}^{p}\left(k\right)$ is determined by the following equation:

(24) $$S_{ff}^{p}\left(k\right)=\frac{\left(S_{max}^{p}-S_{min}^{p}\right)\left(\theta_{ad}\left(k\right)-\theta_{an}\right)}{\theta_{amax}-\theta_{an}}+S_{min}^{p}\;$$

where $\theta_{an}$ is the ankle angle without stimulation (neutral position), and $\theta_{amax}$ is the ankle angle when $S_{max}^{p}$ was applied to the peroneal nerve. The stimulation current to the peroneal nerve at time step *k* by feedback and feed-forward control can be written as shown below:

(25) $$S^{p}\left(k\right)=S_{p}^{p}\left(k\right)+S_{i}^{p}\left(k\right)+S_{d}^{p}\left(k\right)+S_{ff}^{p}\;$$

**Figure 9:**
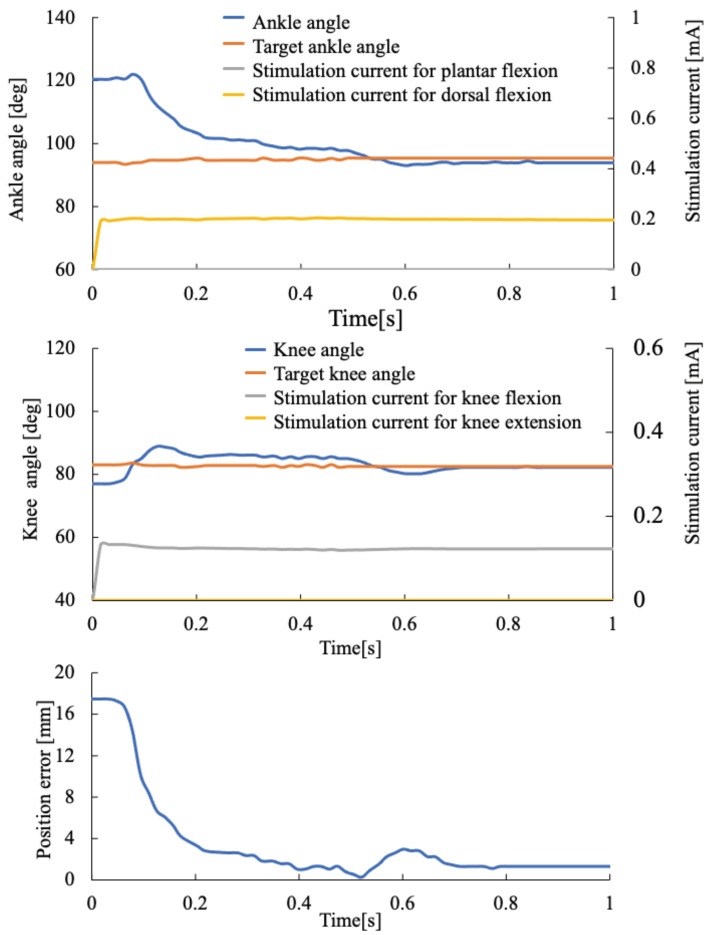
Step response of rat ankle and knee joints when the feedback + feed-forward control was used to move the toe to the target position. The lowest graph indicates the position error of the rat toe during the experiment.

The same procedure was conducted to stimulate the tibial nerve, femoral nerve, and branch of the sciatic nerve connected to the femoral biceps. The step response of rat ankle and knee joints was tested using the feedback + feed-forward control. In the experiment, the parameters for PID control were determined as the same procedure as in previous experiments. The parameters were fixed at $K_{p}$ = 3, $K_{i}$ = 0.3, and $K_{d}$ = 3 in the experiment. The target position of the rat toe was fixed at about 70 degrees from the hip marker and 43% distance from the hip joint to the movable maximum area of the rat toe. Figure 9 shows the results. The knee joint was reached at the target angle for 0.2 s, while the ankle joint was reached at the target angle for 0.35 s. The ankle joint required a longer time to reach the target angle because of the fatigue of the muscle. The tibial anterior muscle, which generates dorsal flexion, is smaller than other muscles for plantar flexion, knee flexion, and extension. Therefore, the response of dorsal flexion was dropped at first when the nerve stimulation was conducted.

**Figure 10:**
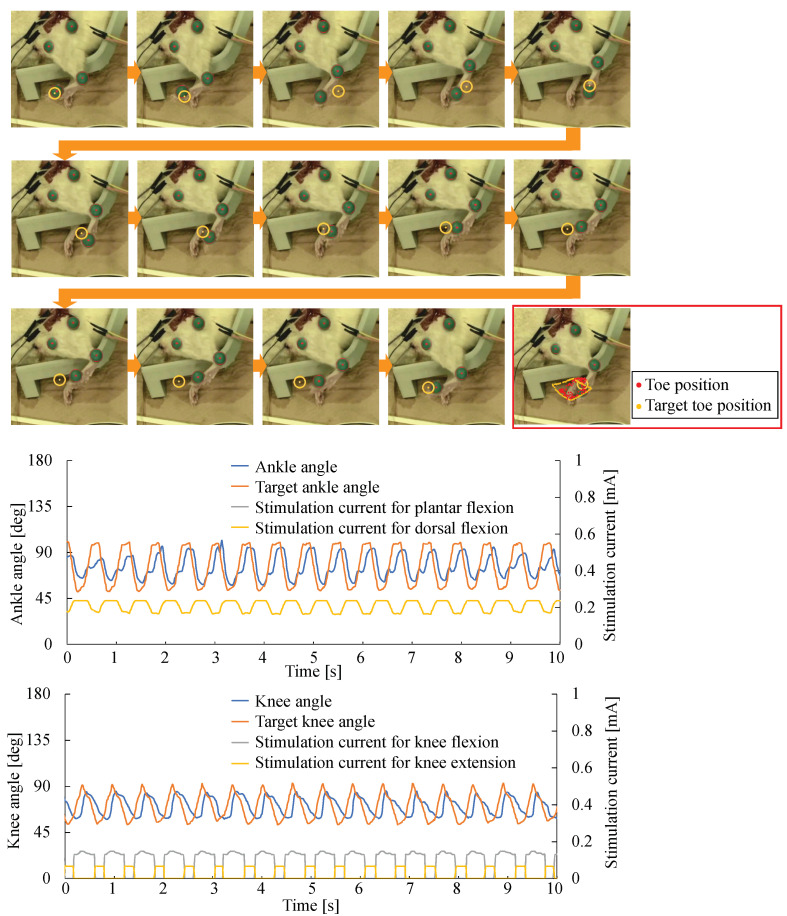
Reconstruction of walking trajectory by ankle and knee joints control. Upper photos indicate the motion of the rat leg during one cycle of the walking trajectory (the orange dot in the orange circle indicates the target position of the rat toe). The right bottom photo (inside the red rectangle) shows the trajectory of the rat toe and the target rat toe in the experiment. Lower graphs show the joint angle, target joint angle, and stimulation currents to the nerves during the experiment.

To reconstruct the walking trajectory of the rat leg, the target trajectory of the rat tow was prepared to mimic the toe motion of a healthy rat walking. The motion was as follows: lift toe and move forward, then down toe to the ground and move backward. The rat was under anesthesia to prevent voluntary movement, and the leg motion was controlled in the air. Therefore, the essential motion of the rat toe was reconstructed in the experiment. The feedback + feed-forward control of ankle and knee joints was conducted to follow the target trajectory of the rat toe. The one cycle of the walking trajectory was set at 1.0 s, which is the same as the walking cycle of a healthy rat. In the experiment, the parameters for PID control were the same as the experiment of the step response. Figure 10 shows the results. Hence, the walking trajectory was reconstructed, although there was a 0.2 s delay from the target trajectory. The result indicates that the feedback + feed-forward control can be used to reconstruct functional motion such as walking, which was a relatively fast motion.

## 4. Discussion

In this study, the four-channel neurostimulator was developed to reconstruct the functional motion of a rat leg. Some neurostimulators have also been developed to generate a muscle contraction. For example, S. H. Cho et al. developed a millimeter-size neurostimulator using Application Specific Integrated Circuit (ASIC) [25]. D. K. Piech also developed a millimeter-scale neurostimulator wirelessly powered using ultrasonic [27]. However, those neurostimulators just generated one muscle contraction by on/off stimulation to a nerve, and the control of stimulation current or leg (or arm) motion was not achieved. A. Shon et al. conducted nerve stimulation during walking of a rat to generate dorsal flexion of the rat ankle [28]. A. D. Mickle et al. conducted control of bladder function in a rat using a wireless closed-loop system [29]. However, the developed system had just one channel, and only dorsal flexion was generated. Hence, conventional neurostimulators generally have only one channel to stimulate one site, and the control of stimulation current or voltage was not precisely controlled. Reconstruction of fast motion by peripheral nerve stimulation was reported by H.J. Park et al. [30], but their study used a large power source and a wired connection between the power source and the nerve. Hence, the proposed method will contribute to reconstructing functional motion for patients with peripheral nerve injury or disorder using a wirelessly powered implantable device.

The position control of the rat leg was achieved by our developed neurostimulator. However, the position control is not enough to reconstruct a higher level of functional motion. For example, force control will be required to reconstruct functional motions such as gripping. The receiver device will be minimized to become implanted into an experimental animal for in vivo test of the developed neurostimulator in the future. Reducing muscle fatigue is also an important issue for neurostimulators. In our experiments, leg motion became smaller even if the stimulation current was the same when the stimulation lasted several minutes. For example, K.T. Gelenitis et al. changed stimulation patterns to the nerve to check muscle fatigue. The sequential stimulation (change the stimulation site in order) showed slower muscle fatigue than the conventional stimulation pattern [31]. R. J. Downey et al. changed the stimulation site on the surface electrodes, and the interleaved stimulation showed lower muscle fatigue than the conventional stimulation [32]. The frequency of electrical stimulation will also be modified to reduce muscle fatigue because the lower frequency may reduce muscle fatigue. Those pattern and frequency changes of stimulation will be employed in our device to reduce muscle fatigue by the electrical stimulation.

## 5. Conclusions

A four-channel wirelessly powered neurostimulator was newly developed to conduct selective FES to the four nerves in a rat. FES was used to generate motion of the ankle and knee joints independently. The neurostimulator used transmitter/receiver coils to wirelessly power the neurostimulator. The receiver device had four outputs, and each output was connected to the four nerves in a rat: the peroneal nerve and tibial nerve for controlling the ankle joint, and the femoral nerve and branch of the sciatic nerve connected to femoral biceps for controlling knee joint. The stimulation of those nerves was switched based on the frequency of the transmitter signal, and the stimulation current was controlled by the amplitude of the transmitter signal. Dorsal and plantar flexion of the rat ankle joint and flexion and extension of the rat knee joint were selectively generated by the developed four-channel neurostimulator. A visual feedback control system was developed to control the toe position by adjusting the angle of the ankle and knee joints. The PID parameters of the visual feedback system were changed, and the two joints were successfully controlled by the developed visual feedback system when the parameters $K_{p}$ = 3 and $K_{i}$ = 0.3 were used. Finally, the walking trajectory was reconstructed by the developed 4-channel neurostimulator. The result indicated that the proposed device and feedback + feed-forward control achieved reconstruction of the walking trajectory of the rat toe.

## Data Availability

Not applicable.
